# Antimicrobial therapy for infected necrotizing pancreatitis: microbiology, antimicrobial resistance and pharmacokinetics

**DOI:** 10.1093/jac/dkag092

**Published:** 2026-03-11

**Authors:** Hannah S Pauw, Rolf Schwarz, Astrid Beij, Elske Sieswerda, Rogier P Voermans, Hjalmar C van Santvoort, Fons F van Den Berg

**Affiliations:** Department of Surgery, St.Antonius Hospital, Nieuwegein, the Netherlands; Department of Research & Development, St.Antonius Hospital, Nieuwegein, the Netherlands; Department of Surgery, St.Antonius Hospital, Nieuwegein, the Netherlands; Department of Research & Development, St.Antonius Hospital, Nieuwegein, the Netherlands; Amsterdam Gastroenterology Endocrinology Metabolism, Amsterdam, the Netherlands; Department of Gastroenterology and Hepatology, Amsterdam UMC, Location University of Amsterdam,Amsterdam, the Netherlands; Julius Centre for Health Sciences and Primary Care, University Medical Center Utrecht, Utrecht University, Utrecht, the Netherlands; European Clinical Research Alliance on Infectious Diseases (Ecraid), Utrecht, the Netherlands; Amsterdam Gastroenterology Endocrinology Metabolism, Amsterdam, the Netherlands; Department of Gastroenterology and Hepatology, Amsterdam UMC, Location University of Amsterdam,Amsterdam, the Netherlands; Department of Surgery, St.Antonius Hospital, Nieuwegein, the Netherlands; Department of Surgery, University Medical Center Utrecht, Utrecht, the Netherlands; Department of Medical Microbiology and Infection Prevention, Amsterdam UMC, Location University of Amsterdam, Amsterdam, the Netherlands; Amsterdam Institute for Immunology and Infectious Diseases, Amsterdam, the Netherlands

## Abstract

Acute pancreatitis is among the most common gastrointestinal disorders requiring hospitalization and can be complicated by serious infections. Approximately 20% of patients progress to necrotizing pancreatitis, of whom ∼30% develop infected pancreatic necrosis, a complication associated with mortality rates of 15%–35% that often necessitates invasive interventions and intensive care treatment. Serious extra-pancreatic infections are also commonly reported in acute pancreatitis patients.

This review summarizes current perspectives on antimicrobial therapy for infected pancreatic necrosis, with an emphasis on microbiology and pharmacokinetics. The microbiological spectrum found in infected pancreatic necrosis is predominantly enteric, reflecting translocation of gut flora into necrotic tissue, with Gram-negative bacteria such as *Escherichia coli* and *Klebsiella* spp. being most frequently isolated. Enterococci and *Candida* species are also commonly identified and have been associated with adverse outcomes, while anaerobes are probably underreported due to inherent culture limitations and antibiotic use. Notably, prolonged hospitalization and cumulative antibiotic exposure select for antimicrobial-resistant and difficult-to-treat pathogens. On the basis of robust evidence, prophylactic antibiotics are not clinically effective in preventing infectious complications. Although carbapenems have traditionally been favoured for treatment of infected pancreatic necrosis on the basis of presumed superior tissue penetration, pharmacokinetic studies suggest that non-carbapenem beta-lactams such as piperacillin-tazobactam and cefepime may achieve adequate tissue penetration as well, particularly when administered as extended infusions. Comparative efficacy studies of antibiotic treatment strategies with clinical endpoints are needed.

## Introduction

Acute pancreatitis is among the most common gastrointestinal disorders requiring hospitalization, with an incidence that has increased by ∼3% annually over recent decades and is now reported at 32.8 cases per 100 000 population (age-standardized rate).^1,2^ Most will have a mild and self-limited disease course, but ∼20% develop necrotizing pancreatitis. Of these, up to 45% will develop organ failure requiring intensive care.^3^ Secondary infection of (peri)pancreatic necrosis (i.e. infected necrosis) occurs in ∼30% and often necessitates invasive intervention. Mortality rates associated with infected necrosis range from 15% to 35%, compared with 0%–1% in mild disease.^4^

The typical disease course of patients with severe acute pancreatitis (SAP) may in theory be divided in an early hyperinflammatory, and late infectious phase.^5^ The first 2 weeks are characterized by systemic inflammatory response syndrome (SIRS) driven by a cytokine storm, followed by a compensatory anti-immune response syndrome that increases susceptibility to infections such as infected necrosis.^6^ Therefore, early mortality is mainly attributed to persistent organ failure due to uncontrolled (sterile) systemic inflammation, while late mortality tends to result from infections. These processes, however, may occur simultaneously, which may explain the observed heterogeneity in the disease course of patients.

Since infected necrosis is a major driver of morbidity and mortality in acute pancreatitis, effective and timely treatment is central to improving outcomes. The cornerstones of management are accurate differentiation between infection and sterile SIRS, prompt initiation of appropriate antibiotics and source control of infected necrosis if needed. However, antimicrobial therapy is typically initiated empirically without microbiological confirmation, and our recent data show that empirically administered regimens are inappropriate in approximately half of patients when compared with subsequent culture results.^7^

## Scope and objectives of the review

This review will summarize the available evidence regarding extra-pancreatic and pancreatic infectious complications in patients with acute (necrotizing) pancreatitis, with a focus on the timing and microbiology of infection, and pharmacokinetic and pharmacodynamic considerations in antibiotic treatment choices. Concomitant biliary infections such as cholecystitis or cholangitis may influence microbial spectrum and antibiotic selection but are not specifically addressed in this review.

## Infectious complications of acute pancreatitis

Peri-pancreatic and parenchymal necrosis can become infected, presumably by translocation of intestinal microbes into necrotic tissue.^8^ The most common source (i.e. upper or lower gastrointestinal tract) and route of infection (i.e. hematogenous, transperitoneal, lymphogenous, reflux into pancreatic duct), however, remains unclear.

Infected necrosis typically develops around 2–4 weeks after disease onset, although 10–15% of patients are diagnosed earlier.^3,9–11^ Patient-level risk factors are limited, with older age and gallstone aetiology showing moderate-to-high certainty of association in a recent meta-analysis.^12^ Other risk factors for infected necrosis largely reflect disease severity, including extent of necrosis, organ failure, and need for mechanical ventilation.^12^ An international consensus-based definition of infected necrosis is the presence of gas configurations within the collection on contrast-enhanced CT and/or positive microbiology result from pancreatic collections,^3^ although clinical criteria such as clinical deterioration despite maximal support may be used for treatment decisions if other causes are excluded.^13^ Current management of infected necrosis centres on two pillars: antimicrobial therapy and source control. In contrast to other abdominal infections, a conservative approach towards source control is needed when managing necrotizing pancreatitis. The most aggressive approach, early open necrosectomy (within 47–72 hours of onset) in patients with necrotizing pancreatitis (either sterile or infected), was associated with a mortality rate of 58%, compared with 27% in the late necrosectomy group (≥12 days of onset).^14^ The combination of (sterile) SIRS and major abdominal surgery most likely led to these high mortality rates. During subsequent decades, a step-approach consisting of (repeated) catheter drainage and minimal invasive necrosectomy performed only in patients with (suspected) infected necrosis became routine care, which was shown to reduce major complications and led to a reduction of late mortality on Dutch ICUs.^15,16^ Surgical and endoscopic interventions demonstrate comparable efficacy, with the advantage of the endoscopic approach being a significantly lower rate of pancreatic fistula formation.^17^ More recently, a Dutch trial by our group showed that delaying source control in patients with (suspected) infected necrosis until necrotic collections were fully encapsulated, while administering appropriate antibiotics as stand-alone therapy was sufficient in ∼40% of patients.^18^ Organ failure and central gland necrosis are the most important predictors for failure of conservative treatment.^7^

## Microbiological spectrum and antimicrobial susceptibility of pancreatic cultures

The source of infecting organisms in infected pancreatic necrosis is predominantly enteric, reflecting translocation of gut flora into necrotic tissue, although molecular analyses indicate that a subset may originate from the oropharynx or airway, particularly in intubated or tracheostomized patients.^19^ Gram-negative bacteria are the dominant group, but the pathogen spectrum evolves over time: early infections are usually caused by enteric pathogens typical for community-acquired intra-abdominal infections, whereas later in the disease course the microbiology increasingly reflects a nosocomial pattern with more antimicrobial-resistant and difficult-to-treat pathogens, probably driven by cumulative antibiotic treatment, prolonged exposure to the hospital environment and introduction of microorganisms through invasive procedures and external pancreatic drains. Table 1 summarizes the identified studies, detailing region, sampling period and pathogen distribution across Gram-negative, Gram-positive, anaerobic and fungal groups.

**Table 1.** Microbiology of infected pancreatic necrosis

First author, year	Country	Period	Population	*N*	Denominator	*K. pneumoniae*	*E. coli*	*A. baumannii*	*P. aeruginosa*	Other Enterobacterales^a^	*E. faecium*	*E. faecalis*	*S. aureus*	CoNS^b^	Streptococci^c^	Anaerobes	*C. albicans*	Non-*C. albicans*
Studies reporting isolate counts																		
Ning 2019	China	2010–2019	IPN	188	504 isolates	82 (16.3%)	55 (10.9%)	65 (12.9%)	32 (6.3%)	50 (9.9%)	59 (11.7%)	10 (2.0%)	13 (2.6%)	13 (2.6%)	17 (3.4%)	4 (0.8%)	31 (6.2%)	30 (6.0%)
Tian 2020	China	2013–2018	SAP	55	58 isolates	6 (10.3%)	5 (8.6%)	13 (22.4%)	4 (6.9%)	NR	9 (15.5%)	—	3 (5.2%)	NR	NR	NR	NR	NR
Garret 2020	France	2012–2015	IPN (ICU)	48	162 species	11 (6.8%)	36 (22.2%)	1 (0.6%)	10 (6.2%)	35 (21.6%)	4 (2.5%)	18 (11.1%)	7 (4.3%)	3 (1.9%)	6 (3.7%)	23 (14.2%)	4 (2.5%)	2 (1.2%)
De Waele 2014	Multinational	2007	SAP (ICU)	159	66 isolates	6 (9.1%)^g^	10 (15.2%)	4 (6.1%)	10 (15.2%)^g^	8 (12.1%)	NR^f^	NR^f^	3 (4.5%)	5 (7.6%)	2 (3.0%)	3 (4.5%)	8 (12.1%)	1 (1.5%)
Studies reporting patient counts																		
Timmerhuis 2023	Netherlands	2010–2019	NP	401	147 patients	5 (3.4%)	48 (32.7%)	—	4 (2.7%)	33 (22.4%)	47 (32.0%)	17 (11.6%)	12 (8.2%)	19 (12.9%)	25 (17.0%)	11 (7.5%)	22 (15.0%)	8 (5.4%)
Würstle 2019	Germany	2005–2016	ANP	122	122 patients	NR	NR	NR	NR	46 (37.7%)^d^	50 (41.0%)	Nr^e^	NR	NR	NR	NR	34 (27.9%)	17 (13.9%)
Huang 2023	China	2010–2022	IPN	294	294 patients	109 (37.1%)	80 (27.2%)	81 (27.6%)	41 (13.9%)	NR	NR	NR	NR	NR	NR	NR	NR	NR
Mowbray 2018	UK	2009–2016	IPN	40	40 patients	2 (5.0%)	8 (20.0%)	—	5 (12.5%)	NR^h^	8 (20.0%)	9 (22.5%)	3 (7.5%)	NR^h^	2 (5.0%)	5 (12.5%)	NR	NR

–, zero; ANP, acute necrotizing pancreatitis; IPN, infected pancreatic necrosis; NP, necrotizing pancreatitis; NR, not reported.

^a^Other *Enterobacterales* includes *Enterobacter*, *Citrobacter*, *Serratia*, *Proteus*, *Morganella*, *K. oxytoca* and *Raoultella* species.

^b^Includes *S. epidermidis*, *S. haemolyticus* and *S. hominis*.

^c^Includes viridans group and other streptococci.

^d^Enterobacteriaceae reported as group.

^e^ *Enterococcus* spp. 64/122 (52.5%), *E. faecium* 50/122 (41.0%).

^f^ *Enterococcus* spp. 15 (22.7%) reported as group.

^g^Reported at genus level.

^h^Isolate counts reported but not per patient: *Enterobacter* (4), *M. morganii* (2), *K. oxytoca* (2), *P. mirabilis* (1), *S. marcescens* (1); CoNS (5), *S. epidermidis* (3), *S. haemolyticus* (1). Upper section: % = isolates/total isolates. Lower section: % = patients with organism/total patients. Only studies with complete microbiological data are shown.

### Gram-negative pathogens

Overall, Gram-negative aerobic bacteria are the most frequently isolated pathogens in infected pancreatic necrosis.^9,20^ In both historical and recent studies, Enterobacterales have featured prominently with *Escherichia coli* being the most common isolate in roughly 20%–35% of cultures.^21–23^ Patterns across cohorts, however, show considerable heterogeneity, which may reflect differences in regional epidemiology, case mix, and timing of sampling rather than true temporal shifts.^9,24^ Other pathogens frequently cultured in infected necrosis include other enteric bacteria such as *Klebsiella* and *Proteus* species, and non-fermenters such as *Pseudomonas aeruginosa* and *Acinetobacter baumannii*.

Early in the course of pancreatic infection, mainly enteric Gram-negatives that are susceptible to first-line antibiotics are found, while later more difficult-to-treat pathogens and carbapenem-resistant pathogens, including Enterobacterales, *P. aeruginosa* and *A. baumannii*, are reported.^19,20,24–26^ The microbiology of infected pancreatic necrosis varies across settings and time periods. A 2009–2016 study from Wales (UK) found that most bacterial pathogens remained susceptible to carbapenems, with only 7% of isolates resistant to meropenem, while a 2019–2023 study from China reported much higher resistance rates, with *Klebsiella pneumoniae* demonstrating carbapenem sensitivity of only 14%–15%.^21,24^ In addition, the *Enterobacter cloacae* complex, which expresses AmpC β-lactamase that hydrolyses most cephalosporins, has been cultured more often in patients who received previous antibiotic therapy, suggesting that early broad-spectrum antibiotics may select for more difficult-to-treat Enterobacterales.^27^ Infections with these nosocomial pathogens are associated with higher morbidity and mortality, although this may reflect confounding by disease severity rather than a direct causal effect.^26^

### Gram-positive pathogens

Enterococci are commonly isolated from infected pancreatic necrosis. In one surgical series, *Enterococcus* spp. were isolated in >20% of cultures in infected pancreatic necrosis cases, although often in the context of polymicrobial infection.^21^ This mirrors other nosocomial intra-abdominal infections and is probably associated with previous antibiotic exposure.^10^ Overuse of broad-spectrum β-lactams in necrotizing pancreatitis may promote intestinal overgrowth of enterococci, facilitating translocation into necrotic pancreatic tissue.^10,28^  *Enterococcus* spp. are difficult to treat due to limited susceptibility to antibiotics. In one observational study, detection of *Enterococcus faecium* was associated with a >3-fold increase in odds of death (OR 3.73, 95% CI 1.38–10.05, *P* = 0.009).^10^ Similarly, a recent Dutch multicentre study found that the development of an enterococcal infection was strongly associated with worse outcomes. This study confirmed that prolonged antibiotic exposure was a risk factor for acquiring *Enterococcus* spp. (OR 1.08 per day of therapy, 95% CI 1.03–1.16, *P* = 0.01), and infection with *Enterococcus* spp. was itself linked to higher rates of persistent organ failure (OR 3.08, 95% CI 1.35–7.29, *P* < 0.01) and mortality (OR 5.78, 95% CI 1.46–38.73, *P* = 0.03).^28^ Again, causality of *Enterococcus* detection with adverse outcomes remain uncertain. In addition, in case of vancomycin-resistant enterococci (VRE), there is an increased risk of treatment failure and death due to unavailability of other antibiotic options.

*Staphylococcus aureus* is a less common cause of infected pancreatic necrosis, although they are detected in 7%–8% of infected (peri)pancreatic collections.^28,29^ When staphylococci do appear, it is often in the context of extra-pancreatic infection (for example, catheter-related bloodstream infection or ventilator-associated pneumonia) rather than as a primary organism in necrotic pancreatic tissue.^10^ Coagulase-negative staphylococci are occasionally reported as well, but these isolates are generally considered more likely to represent procedural contamination than true infection. *S. aureus* (including methicillin-resistant *S. aureus*) can complicate the course of patients with severe acute pancreatitis, but it remains an infrequent isolate from pancreatic necrosis itself.

Despite their abundance in the upper gastrointestinal tract, Streptococcus species are uncommonly isolated from infected collections, with prevalence varying between cohorts (3%–17%, Table 1). Molecular analysis suggests this low prevalence reflects true rarity rather than under-detection due to previous antibiotic exposure.^19,30–33^

### Anaerobic pathogens

Anaerobic pathogens often contribute to polymicrobial infected collections, however, their detection in infected necrosis varies significantly. For example, a study from the UK reported anaerobes in 12.5% of infected pancreatic necrosis cases, while a large Chinese cohort identified Bacteroides species in <1% of all isolates.^21,34^ Additional culture-based cohorts confirm the presence of anaerobic genera such as *Bacteroides*, *Clostridium*, *Prevotella*, *Fusobacterium*, and *Peptostreptococcus*, although without quantification.^10^ However, their true prevalence during the early disease course of infected pancreatic necrosis is probably underestimated because of the inherent difficulties in culturing anaerobes and cumulative antibiotic administration before sample collection. A small, retrospective study at the ICU showed high culture yields in infected necrosis cultures in both patients with and without previous antibiotics (81% and 69.5%, respectively), however, fewer anaerobes were isolated among pretreated patients.^27^ The higher total yield in pretreated patients is suggestive for selection bias, limiting interpretability of the findings. Their true prevalence is probably underestimated due to inherent difficulties in culturing anaerobes and cumulative antibiotic exposure before sampling; culture-independent molecular studies (discussed later) confirm substantially higher detection rates.^10,19^

### Fungal infections

Fungal infection of pancreatic necrosis, almost always by *Candida* spp., is a prognostic and therapeutic important complication of acute pancreatitis. Recent evidence shows a substantial incidence of pancreatic fungal infection, with a mean incidence of 26.6% in necrotizing pancreatitis patients from a meta-analysis of 22 studies.^35^  *Candida albicans* is the most frequently identified species.^20,28^ Non-albicans species, including *C. glabrata*, *C. tropicalis* and *C. parapsilosis*, account for ∼30%–50% of *Candida* isolates in available cohorts (Table 1). Fungal infection has been linked to higher morbidity and mortality in multiple studies. The meta-analysis found that patients with pancreatic fungal infection had nearly four times higher odds of death than those without (pooled OR 3.95, 95% CI 2.6–5.8).^35^ In particular, infections by non-albicans *Candida* spp. are concerning, as these species may exhibit resistance to fluconazole and other azole antifungals.^10^ The presence of non-albicans *Candida* spp. in infected pancreatic necrosis has been noted as a marker of especially severe or prolonged infection, carrying an even higher risk of death in one study (OR 3.32, 95% CI 1.07–10.35, *P* = 0.039).^10^ Peri-procedural contamination of the sample during endoscopic drainage procedure, especially in highly colonized patients with prolonged antibiotic therapy, is possible and may bias these findings.

### Molecular analyses

Molecular techniques reveal higher pathogen detection rates than culture in infected pancreatic necrosis. Four studies comparing metagenomics with culture from (peri)pancreatic specimens found 162 versus 91 total isolates (Table 2).^30–33^ A 16S rRNA sequencing study detected anaerobes in 30/56 (53.6%) pancreatic samples—*Bacteroides fragilis* in 29%, *Dialister invisus* and *Olsenella uli* each in ∼13%—yet none were captured by routine culture.^19^ Similarly, metagenomics detected anaerobes in 10%–18% of isolates whereas culture detected none across all four studies. For aerobic pathogens (*E. coli*, *K. pneumoniae*, *A. baumannii*, *E. faecium*), detection rates were comparable between methods. These findings confirm the under-detection of anaerobes and support empirical anaerobic coverage despite low culture yields.

**Table 2.** Pathogens detected by mNGS versus culture in (peri)pancreatic specimens from infected pancreatic necrosis

First author, year	Country	Sample type	Method	Denominator	*K. pneumoniae*	*E. coli*	*A. baumannii*	*P. aeruginosa*	Other Enterobacterales^a^	*E. faecium*	*E. faecalis*	*S. aureus*	CoNS	Anaerobes^b^	*Candida* spp.
Hong 2022	China	Pancreatic fluid	mNGS	38 isolates	3 (7.9%)	4 (10.5%)	4 (10.5%)	2 (5.3%)	5 (13.2%)	6 (15.8%)	1 (2.6%)	—	1 (2.6%)	7 (18.4%)	—
			Culture	24 isolates	4 (16.7%)	4 (16.7%)	5 (20.8%)	1 (4.2%)	1 (4.2%)	5 (20.8%)	—	—	1 (4.2%)	—	2 (8.3%)
Hong 2024	China	FNA	mNGS	16 isolates	7 (43.8%)	1 (6.3%)	3 (18.8%)	—	1 (6.3%)	2 (12.5%)	—	—	2 (12.5%)	—	—
			Culture	18 isolates	7 (38.9%)	1 (5.6%)	3 (16.7%)	—	1 (5.6%)	2 (11.1%)	—	—	2 (11.1%)	—	2 (11.1%)
Zhang 2025	China	Pancreatic fluid	mNGS	39 isolates	3 (7.7%)	10 (25.6%)	4 (10.3%)	1 (2.6%)	2 (5.1%)	4 (10.3%)	3 (7.7%)	1 (2.6%)	3 (7.7%)	—	3 (7.7%)
			Culture	28 isolates	4 (14.3%)	5 (17.9%)	4 (14.3%)	1 (3.6%)	1 (3.6%)	2 (7.1%)	4 (14.3%)	1 (3.6%)	2 (7.1%)	—	2 (7.1%)
Lin 2022	China	Peri-pancreatic	mNGS	70 isolates	17 (24.3%)	5 (7.1%)	15 (21.4%)	3 (4.3%)	13 (18.6%)	7 (10.0%)	—	2 (2.9%)	1 (1.4%)	7 (10.0%)	—
			Culture	21 isolates	5 (23.8%)	2 (9.5%)	3 (14.3%)	1 (4.8%)	2 (9.5%)	6 (28.6%)	—	1 (4.8%)	1 (4.8%)	—	—

–, not detected; FNA, fine-needle aspiration; mNGS, metagenomic next-generation sequencing.

^a^Other *Enterobacterales* includes *Enterobacter*, *Citrobacter*, *Serratia*, *Klebsiella aerogenes*, *Salmonella*.

^b^Anaerobes: *Bacteroides*, *Peptostreptococcus*, *Prevotella*, *Finegoldia*, *Anaerococcus*, *Clostridium*.

## Spatiotemporal distribution of antimicrobial resistance

Susceptibility patterns of pathogens isolated from infected necrotic collections highly vary by region and over time. A Chinese retrospective study of 188 patients with infected pancreatic necrosis demonstrated that isolation of multidrug-resistant organisms (MDRO) from 2010 to 2019 increased from 16.7% to 74.6% of patients.^34^ A German multicentre study demonstrated a substantial reduction in Enterobacteriaceae susceptibility for reserve antibiotics over 100 days of hospitalization, isolated from pancreatic punctures (87% susceptible early versus. only 27% susceptible late).^10^ In parallel, the cumulative incidence of MDRO isolation rose steadily, with nearly 50% of patients infected with MDRO after 150 days of hospitalization. Similarly, another study reported that half of ICU patients with infected pancreatic necrosis had an MDR or extremely drug-resistant pathogen identified at some point during treatment.^27^ The high prevalence of MDR infection in late pancreatitis is also confirmed by a recent large study. A Chinese study found an 87.6% rate of MDR organisms among cultured pancreatic infections in AP, with carbapenem-resistant *K. pneumoniae*, *P. aeruginosa*, *A. baumannii* and *E. faecium* being the most prevalent.^24^ An earlier study from India likewise showed that among acute pancreatitis patients who developed infections, 87% developed an MDR bacterial infection at some point and nearly 50% acquired an extremely drug-resistant organism (resistant to almost all classes) during their course.^36^ Notably, these very high MDRO rates from China and India reflect high endemic antimicrobial resistance in these regions and may not be directly generalizable to settings with lower baseline resistance. MDROs and other difficult-to-treat pathogens such as fungi and enterococci are associated with ongoing antibiotic pressure and prolonged hospitalization, which creates a selective environment favouring the emergence of multidrug-resistant pathogens in later phases of disease.

## Prophylactic antibiotics

Early studies in the 1980s–1990 s demonstrated that prophylactic broad-spectrum antibiotics, particularly imipenem, might reduce infection rates and mortality in patients with acute necrotizing pancreatitis.^37–39^ Consequently, these findings were included in the guidelines, and prophylactic antibiotics were frequently used in clinical practice. However, subsequent randomized controlled trials failed to replicate these findings. Two high-quality double-blind RCTs that investigate prophylactic ciprofloxacin and metronidazole (*n* = 114) and meropenem (*n* = 100), initiated antibiotics early (≤72–120 hours) using strict criteria for predicted severe or confirmed necrotizing pancreatitis, and found no reduction in infected necrosis (12% versus 9%, *P* = 0.46% and 18% versus 12%, *P* = 0.40, respectively), mortality (5% versus 7%, *P* = 0.76% and 20% versus 18%, *P* = 0.80, respectively) or need for surgical intervention (26% versus 20%, *P* = 0.48, respectively).^40,41^ Following these conflicting results, multiple meta-analyses have evaluated prophylactic antibiotics in necrotizing or severe acute pancreatitis.^42–48^ Some meta-analyses performed a pooled analysis regardless of antibiotic regimens that varied from fluoroquinolone-based combinations, cephalosporins and imipenem. Other meta-analyses focused solely on carbapenems or performed subgroups analyses.^42–44,46,47^ Despite these methodological differences, the overarching conclusions have been consistent: prophylactic antibiotics do not significantly reduce infected necrosis or mortality. This has been adopted in more recent guidelines and this practice has been banned from routine clinical care.^49–53^ Likewise, routine anti-fungal prophylaxis is not advised, as historical recommendations were based on limited and mixed evidence and therefore current guidelines do not recommend its use.^49–57^

## Pharmacokinetic and pharmacodynamic considerations

Early studies showed high penetration of imipenem in pancreatic tissue, and a benefit of prophylactic treatment, led to the widespread use of carbapenems for the treatment of (suspected) infected necrotizing pancreatitis.^58,59^ This was also adopted in many guidelines.^49–52^ Some guidelines do caution against routine empirical carbapenem use, advising that their use should be used restrictively in critically ill patients.^53^

Considerations for empirical antibiotic regime to treat patients with infected necrotizing pancreatitis includes the antimicrobial spectrum, local antimicrobial resistance levels, pharmacokinetics and pharmacodynamics. In addition, patient-related factors, disease course, antibiotic exposure and previous culture results may be of influence. Comparative clinical data are limited to a single small retrospective study (*n* = 63) comparing meropenem with piperacillin-tazobactam, which found no significant difference in 90-day clinical failure but a higher infection recurrence rate with piperacillin-tazobactam; however, no firm conclusions can be drawn due to the study's inherent limitations including baseline differences between groups.^60^

On the basis of the antimicrobial spectrum of infected necrosis cultures, empirical therapy in the early phase of infected pancreatic necrosis diagnosis should at least include coverage of frequently cultured Gram-negative bacteria, such as *E. coli* and most *Klebsiella* species, as these species are most frequently found in infected tissues. Most of these strains are sensitive to among others second- and higher-generation cephalosporins, piperacillin-tazobactam and carbapenems, depending on the local resistance epidemiology. The extent to which anaerobes contribute to infected necrosis remains unclear, however, on the basis of the pathophysiology of bacterial translocation, anaerobic coverage seems imperative. Enterococci are frequently cultured in infected pancreatic necrosis, however, their clinical significance varies by disease phase. At initial presentation of infected pancreatic necrosis without previous antibiotic exposure, enterococcal coverage is generally not recommended given limited evidence of benefit in community-acquired intra-abdominal infections with adequate source control.^61^ In recurrent or ongoing infected pancreatic necrosis—particularly in patients with substantial previous broad-spectrum antibiotic exposure or previous enterococcal cultures—anti-enterococcal therapy may be considered on an individual basis. Empiric anti-fungal coverage is not recommended without documented or strongly suspected fungal infection.^62,63^ Instead, therapy may be considered if *Candida* species are isolated from pancreatic cultures or strongly suspected based on clinical features, although clinical efficacy data are lacking.

The necrotic pancreas is a unique and challenging site for antibiotic delivery. Necrotic collections have areas of poor blood flow, high proteolytic enzyme activity and, in some cases, a fibrous wall if walled-off necrosis has developed.^3^ These factors can impair antibiotic penetration and, once extensive necrosis has developed, penetration becomes more difficult as active tissue perfusion is limited. Consistent with this, antibiotic concentrations in necrotic pancreatic tissue are generally lower than those achieved in normal and oedematous pancreatic tissue.^64^ Antibiotics with small molecular size, low protein binding and good diffusion tend to penetrate better (e.g. beta-lactams), in contrast to large-molecule antibiotics.^65^ From a pharmacologic perspective, beta-lactams are time-dependent agents, and the duration that free drug levels remain above the minimum inhibitory concentration (f*T* > MIC) is the critical determinant of efficacy, especially in patients with SIRS or sepsis.^66^

Historically, carbapenems, specifically imipenem, were thought to have superior penetration into the pancreatic tissue.^58^ However, a later study restricted to acute necrotizing pancreatitis found substantially lower intrapancreatic concentrations than the earlier data suggested.^67^ More recent evidence indicates that other beta-lactams also penetrate sufficiently.^65^ Piperacillin-tazobactam, for example, achieves high intra-necrotic concentrations—comparable to plasma levels—when administered intermittently, indicating substantial penetration into necrotic pancreatic tissue.^68^ Third- and fourth-generation cephalosporins also penetrate pancreatic tissue.^65^ Ceftriaxone and cefotaxime achieve tissue homogenate concentrations of ∼6 and 9 mg/kg, respectively; although these values cannot be directly compared to MICs, ceftriaxone’s long half-life (5–9 hours) supports adequate target attainment.^58,69^ Metronidazole, often combined with cephalosporins for anaerobic coverage, also achieves adequate pancreatic tissue concentrations (3.5–8.5 mg/kg in tissue homogenate) due to its small molecular size and extensive tissue distribution.^58,67^ Cefepime demonstrates lower pancreatic penetration with mean tissue concentrations of ∼10 mg/L after a 2 g dose, sufficient for organisms with low MICs.^65^ PK/PD modelling indicates that achieving 60%–70% f*T* > MIC for less susceptible pathogens may require more aggressive dosing or extended infusions, but target attainment remains feasible overall. In critically ill patients, standard PK/PD targets may be insufficient; higher targets such as 100% f*T* > MIC or 100% f*T* > 4×MIC have been associated with improved clinical outcomes and may be warranted in this setting.^70,71^ Fluoroquinolones, more specifically ofloxacin, were used in combination with metronidazole as an alternative to carbapenems in older studies. The best parameters for target attainment of fluoroquinolones is the AUC/MIC PK/PD (optimal target 125–250), but available data show low and inconsistent pancreatic tissue concentrations, making target attainment increasingly difficult as MICs rise.^65^ Combined with safety concerns (tendinopathy) and increasing resistance, their PK/PD profile makes them a suboptimal empirical choice especially in critically ill patients, however, it may be used for step-down antibiotic treatment. Aminoglycosides (e.g. gentamicin) have poor tissue penetration including into the pancreas and are nephrotoxic.^67^ Therefore, they are not recommended as monotherapy but may be considered as adjunctive therapy in patients with septic shock.^72,73^ Vancomycin is frequently added to the regime when ampicillin-resistant enterococci are identified in pancreatic cultures, however, due to its large molecule size, tissue distribution may not be sufficient to treat pancreatic infections.^74^ In settings with higher VRE prevalence, linezolid may be an alternative given its smaller molecular size and low protein binding. Limited data suggest adequate penetration into pancreatic secretions.^75^ Limited data also show that fluconazole may sufficiently penetrate the pancreas, with pancreatic concentrations that are well above the average MIC of most commonly identified *Candida* species.^76^ No pharmacological studies with echinocandins have been published.

In critically ill patients, increased volume of distribution and augmented clearance may result in underdosing with standard regimens. In an ICU setting it is therefore often recommended to do extended or continuous infusions of beta-lactams to maximize tissue concentrations, although randomized data have not demonstrated a definitive mortality benefit, and the superiority of continuous infusion remains debated.^65,77^ For necrotizing pancreatitis, it was suggested that standard intermittent dosing of meropenem (e.g. 1 g every 8 h, 30-min infusions) might not reliably achieve target levels against *Pseudomonas*, as demonstrated by Monte Carlo simulations.^65,78^

## Recommendations

Recommendations for the choice of empirical antibiotics for (suspected) infected necrosis in patients with acute pancreatitis are highly dependent on: (i) antimicrobial spectrum and resistance, (ii) pharmacokinetics and (iii) previous antibiotic administration and known cultures. Given that empirical regimens are insufficient in approximately half of patients, culture-guided treatment should be pursued when feasible. With current trends towards delayed intervention and antibiotic-only treatment strategies, fewer patients undergo drainage procedures, limiting the availability of cultures to guide therapy. Routine fine-needle aspiration has fallen out of practice due to limited added benefit to diagnose infected necrosis, however, it may be considered before initiating antibiotics to enable targeted antimicrobial treatment.

The available literature does not support superiority of either carbapenems or carbapenem-sparing alternatives such as cefepime and piperacillin-tazobactam in terms of penetration of (infected) pancreatic tissue. A combination of a cephalosporin (second generation or higher) and metronidazole may be adequate in countries with low-level resistance such as the Netherlands. Piperacillin-tazobactam may be considered in high-level resistance settings or if coverage of enterococci is deemed necessary on the basis of on the patient’s history or severity of illness. Carbapenems and other last-line antibiotics may be reserved for infections with multi-drug-resistant organisms or in case of clinical failure of first-line therapy, thereby reducing selective pressure that drives carbapenem-resistant and MDRO infections. Anaerobes and streptococci are probably underreported and empirical coverage is recommended. Empirical anti-fungal treatment is currently recommended against, despite positivity rates of 10%–20% in pancreatic cultures and association with worse outcome. It may be considered depending on the duration of hospitalization, antibiotic exposure and treatment failure with broad-spectrum therapy.

Antibiotic regimen adjustment following culture results is mainly useful to broaden the antibiotic spectrum in case of resistant Enterobacterales, yeast, and/or enterococci. When deciding to adjust the antibiotic regimen, differentiating between infection and (drain) colonization is essential. Cultures should be obtained during or soon after the drainage procedure, as later drain cultures are uninterpretable and may lead to overtreatment. Enterococci and yeast recovered from drain fluid several weeks after placement often represent colonization rather than invasive disease. Duration of antimicrobial therapy should be individualized based on clinical response, timing and adequacy of source control, and differentiation between active infection and colonization, as definitive evidence on optimal treatment duration is lacking.
